# The Teeth in Diabetes

**Published:** 1883-06

**Authors:** 


### THE TEETH IN DIABETES.

Dr. Magitot has, after an examination of many diabetics, come to the following conclusions: First, examination of the mouth of diabetics furnishes a constant symptom of the disease. Second, this symptom is a lesion of the alveolar border, which may be dedesignated as an alveolar osteo-periostitis. Third, this manifestation appears at the outset of the disease, persists during its course, and can, in consequence be considered a pathognomonic symptom. Fourth, this alveolar affection considered as a symptom of diabetis, presents three periods. Its first period is that of simple deviation of the teeth. Its second period is that of loosening of the teeth and alveolar catarrh. Each of these periods is in relation to the phase of the constitutional disease. The third period, that of the falling out of the teeth, corresponds to a more advanced state of glycosuria. Besides this last symptom there may occur, if the patient lives long enough, an osseous resorption, which may or may not be consecutive to a gangrene of the gums. The appearance of this latter complication is evidence of a critical stage of the disease, as it ordinarily ushers in its fatal termination. The value, as a symptom, of the first stage of dental changes, remains to be determined. It must be obvious, however, that it can only occur in the more chronic forms of glycosuric diabetis.

